# The Comparative Methylome and Transcriptome After Change of Direction Compared to Straight Line Running Exercise in Human Skeletal Muscle

**DOI:** 10.3389/fphys.2021.619447

**Published:** 2021-02-19

**Authors:** Mohd-Firdaus Maasar, Daniel C. Turner, Piotr P. Gorski, Robert A. Seaborne, Juliette A. Strauss, Sam O. Shepherd, Matt Cocks, Nicolas J. Pillon, Juleen R. Zierath, Andrew T. Hulton, Barry Drust, Adam P. Sharples

**Affiliations:** ^1^Stem Cells, Aging and Molecular Physiology Unit, Exercise Metabolism and Adaptation Research Group, Research Institute for Sport and Exercise Sciences, Liverpool John Moores University, Liverpool, United Kingdom; ^2^Institute for Science and Technology in Medicine, School of Pharmacy and Bioengineering, Keele University, Staffordshire, United Kingdom; ^3^Institute for Physical Performance, Norwegian School of Sport Sciences, Oslo, Norway; ^4^Centre for Genomics and Child Health, Blizard Institute, Barts and the London School of Medicine and Dentistry, Queen Mary University of London, London, United Kingdom; ^5^Exercise Metabolism and Adaptation Research Group, Research Institute for Sport and Exercise Sciences, Liverpool John Moores University, Liverpool, United Kingdom; ^6^Department of Physiology and Pharmacology, Karolinska Institutet, Stockholm, Sweden; ^7^Department of Molecular Medicine and Surgery, Karolinska Institutet, Stockholm, Sweden; ^8^Novo Nordisk Foundation Center for Basic Metabolic Research, University of Copenhagen, Copenhagen, Denmark; ^9^Department of Nutritional Sciences, School of Biosciences and Medicine, Faculty of Health and Medical Sciences, University of Surrey, Guildford, United Kingdom; ^10^School of Sport, Exercise and Rehabilitation Sciences, College of Life and Environmental Sciences, University of Birmingham, Birmingham, United Kingdom

**Keywords:** DNA methylation, VEGF, change of direction, PGC1 alpha, NR4A1 (Nur77), NR4A3, AMPK, MAPK

## Abstract

The methylome and transcriptome signatures following exercise that are physiologically and metabolically relevant to sporting contexts such as team sports or health prescription scenarios (e.g., high intensity interval training/HIIT) has not been investigated. To explore this, we performed two different sport/exercise relevant high-intensity running protocols in five male sport team members using a repeated measures design of: (1) change of direction (COD) versus; (2) straight line (ST) running exercise with a wash-out period of at least 2 weeks between trials. Skeletal muscle biopsies collected from the vastus lateralis 30 min and 24 h post exercise, were assayed using 850K methylation arrays and a comparative analysis with recent (subject-unmatched) sprint and acute aerobic exercise meta-analysis transcriptomes was performed. Despite COD and ST exercise being matched for classically defined intensity measures (speed × distance and number of accelerations/decelerations), COD exercise elicited greater movement (GPS-Playerload), physiological (HR), metabolic (lactate) as well as central and peripheral (differential RPE) exertion measures compared with ST exercise, suggesting COD exercise evoked a higher exercise intensity. The exercise response alone across both conditions evoked extensive alterations in the methylome 30 min and 24 h post exercise, particularly in MAPK, AMPK and axon guidance pathways. COD evoked a considerably greater hypomethylated signature across the genome compared with ST exercise, particularly at 30 min post exercise, enriched in: Protein binding, MAPK, AMPK, insulin, and axon guidance pathways. Comparative methylome analysis with sprint running transcriptomes identified considerable overlap, with 49% of genes that were altered at the expression level also differentially methylated after COD exercise. After differential methylated region analysis, we observed that VEGFA and its downstream nuclear transcription factor, *NR4A1* had enriched hypomethylation within their promoter regions. *VEGFA* and *NR4A1* were also significantly upregulated in the sprint transcriptome and meta-analysis of exercise transcriptomes. We also confirmed increased gene expression of *VEGFA*, and considerably larger increases in the expression of canonical metabolic genes *PPARGC1A (that encodes PGC1-*α) and *NR4A3* in COD vs. ST exercise. Overall, we demonstrate that increased physiological/metabolic load via COD exercise in human skeletal muscle evokes considerable epigenetic modifications that are associated with changes in expression of genes responsible for adaptation to exercise.

## Introduction

Epigenetic modifications, particularly DNA methylation, are associated closely with altering gene expression in response to exercise in human skeletal muscle (Turner et al., 2019). Genome-wide DNA methylation analysis after acute resistance exercise, training, detraining and retraining in humans has demonstrated that epigenetic modifications can be retained and therefore skeletal muscle possesses an epigenetic memory (Seaborne et al., 2018a). Resistance exercise preferentially reduces or hypomethylates the DNA methylome (Seaborne et al., 2018a, b) and increases gene expression across the transcriptome (Turner et al., 2019). Further, even greater hypomethylation was observed if exercise training had been undertaken previously, such as following retraining (Seaborne et al., 2018a, b). For example, the candidate gene ubiquitin protein ligase E3 component N-recognin 5 (UBR5) demonstrating this epigenetic profile, has now been determined as an important regulator of skeletal muscle mass (Seaborne et al., 2019; Hughes et al., 2021). Therefore, alterations in DNA methylation signatures are emerging as important drivers in the molecular response to exercise and subsequent physiological adaptation.

Methylation of DNA occurs most commonly in CpG sites. CpG islands are CpG rich locations that occur more frequently in gene regulatory regions, such as promoters. In humans the majority of CpG sites are methylated (70–80%) (Lister et al., 2009; Ziller et al., 2013). When methylation is present or increases (hypermethylation), particularly in promoters, this generally prevents transcription factors from binding to these portions of the gene to enable gene expression (Bogdanovic and Veenstra, 2009). In contrast, loss of methyl groups (hypomethylation) from these regions generally enables/increases gene expression.

Aerobic exercise training alters the skeletal muscle methylome in humans (Lindholm et al., 2014) and preferentially hypomethylates the genome in type-II diabetes (Nitert et al., 2012) and obese populations (Rowlands et al., 2014). At the targeted gene level, acute aerobic exercise, particularly at higher intensities (80% vs. 40% VO_2_ max) hypomethylates *PPARGC1A* that encodes *PGC-1*α, a well-established regulator of mitochondrial biogenesis (Handschin et al., 2007). The same trend is also observed for associated metabolic pathway genes; *TFAM, PDK4*, and *PPARD* (Barres et al., 2012), together with a corresponding increase in their gene expression (Barres et al., 2012). However, there is limited analysis of DNA methylation across the genome after acute aerobic exercise in healthy human skeletal muscle. Furthermore, there is currently no temporal assessment of the methylome post-acute aerobic exercise, such as after 24 h or after alterations in exercise intensity. Finally, the methylome signature following exercise that is physiologically and metabolically relevant to sporting contexts such as team sports or health prescription scenarios (e.g., high intensity interval training/HIIT) in the general public has not been investigated.

In the present study, we devised a repeated measures sprint shuttle running protocol in humans that was physiologically relevant to exercise movements within many sporting contexts and health prescription settings. In the design there were two different sprint shuttle running trials: (1) change of direction (COD) and, (2) straight line (ST) running exercise that were both matched for classically defined intensity measures (speed × distance) and the number of acceleration and deceleration movements. However, despite this matched intensity between trials, we hypothesized that the COD exercise protocol would evoke increases in movement (GPS Playerload^TM^), physiological (HR), metabolic (lactate), subjective central and peripheral (differential RPE) measures. Therefore, the comparison of COD versus ST exercise would provide a suitable model to investigate DNA methylome changes following increased loading exercise in human skeletal muscle in a physiologically relevant model. Consequently, we first aimed to investigate differential DNA methylation across the genome between these two trials with additional dedicated genome-wide analyses for enriched gene ontologies, pathways and chromosomal regions 30 min and 24 h after COD compared with ST exercise. Furthermore, using the methylome data derived in the present study, we undertook integrated comparative analysis with recently published transcriptome data taken after acute sprint exercise in humans (Rundqvist et al., 2019), and also overlapped the methylome with recent meta-analysis that combines transcriptome data from 66 published data sets in the most comprehensive exercise transcriptome profiling to date (Pillon et al., 2020). Subsequently, the second aim was to determine if the alterations across the methylome between COD versus ST exercise were also seen at the transcriptome level. Finally, based on genes identified at both the methylome (site and region level) and transcriptome level we aimed to confirm if gene expression was altered via quantitative RT-PCR at the individual gene level. Given early DNA methylation changes precede gene expression (Seaborne et al., 2018a, b) and gene expression typically peaks at 3–6 h post exercise, we also determined gene expression at 3 h (in addition to 30 min and 24 h) post exercise in both COD and ST trials. We hypothesized that increased loading observed in the COD compared with ST exercise trial would evoke a greater hypomethylated signature across the epigenome, with this response being validated in predominantly the same genes at transcript level after comparative analysis with recently published exercise transcriptomes. The ultimate aim of the study was to identify important epigenetic regulators in the response to acute exercise in human skeletal muscle.

## Materials and Methods

### Ethics Statement

Ethical approval was granted from the institutional ethics review board/committee of Liverpool John Moores University (ref. 19/SPS/028). All methods were performed in accordance with the relevant guidelines and regulations of this review board.

### Experimental Overview

Following preliminary visits to the laboratory to complete screening and pre-test assessments/familiarization, a repeated measures design was used for all participants to complete two experimental trials; a 180-degree change of direction (COD) performed over a distance of 5 m to volitional exhaustion and a repeated straight line (ST) 5 m sprint protocol (ST) performed at the same intensity (running speed) until an equivalent distance to that covered in COD was completed (3.2 ± 0.1 m.s^−1^; distance: 594 ± 35 m). The individual responses to these exercise bouts were described from a movement (GPS player load), physiological (HR), metabolic (lactate) and subjective perspective (central and peripheral fatigue/rating of perceived exertion – RPE) to holistically characterize the ‘load’ demand associated with the activity. Muscle biopsies were obtained from the lateral portion of the vastus lateralis muscle for genome wide DNA methylation analysis, comparative transcriptome and targeted gene expression, described in detailed below.

### Participants

A group of five healthy, male team sports players (age: 26 ± 2 years; height: 1.7 ± 0.1 m; weight: 76.3 ± 11.5 kg; minimum of three training sessions per week) were recruited to participate in this study. After giving informed consent, all participants were screened using physical activity and readiness to exercise questionnaires and a pre-biopsy medical assessment. The information was used by the research team and a qualified medical practitioner to confirm inclusion/exclusion criteria and eligibility for the current study. All participants included were free from any musculoskeletal injuries for the last 6 months. Participants were required to refrain from strenuous exercise 24 h prior to testing, alcohol for 48 h prior to exercise and caffeine for 12 h prior to exercise.

### Pre-testing Assessments/Familiarization

#### Max Running Speed Test

All participants attended the laboratory, a maximum of two occasions, to complete a pre-test assessment to determine the running speed to be used in the experimental protocols and to complete familiarization with the relevant measurement tools. Each participant was required to perform a 6 × 5 m shuttle run with a 180-degree COD at their maximal speed. These runs were performed until the speed at which the shuttles were completed demonstrated a coefficient of variation of 5% or lower. This intensity was also re-evaluated on a second visit to ensure that participants could re-produce the required running speed identified in the first session to the same accuracy (3.21 ± 0.1 m.s^–1^). This speed was then used for the completion of all subsequent shuttle runs, programmed into a computer derived audible beep. All participants also performed 10 repetitions in a preliminary trial of COD with 30-s rest in between repetitions. This acted as familiarization to both the movement patterns required for the protocol and the approach to be used to evaluate the subjective responses to exercise.

#### Experimental Exercise Protocols

The same participants performed two experimental protocols following an overnight fast; COD and ST running trials on separate occasions separated by a minimum of 2 weeks. All participants completed the COD protocol followed by the ST trial, this was because the COD trial was until voluntary exhaustion and the ST trial was then matched for the same repetitions and distance in the COD trial, described fully below. Both protocols were preceded by the completion of a standardized 10 min warm-up which consisted of 5 min slow running to elevate HR. This activity was followed by dynamic and static stretching to increase range of movement and readiness for the specific movement pattern of the running protocol. The warmup was led by the researcher. The intensity (running speed) of COD was controlled using a computer derived audible beep determined based on the maximum individual speed collected during the pre-test assessment. After each repetition (6 × 5 m runs separated by a 180° COD), a 30 s rest period was completed. COD was performed until voluntary exhaustion (defined as the point at which the participant could no longer complete the required shuttle distance at the pre-determined speed). The ST sprints were performed at an equivalent intensity to that used in the COD trial. ST sprints required individuals to perform repeated 5 m sprints in a forward direction over a 30 m course. To maintain a similar acceleration and deceleration profile to that of the COD participants were required to come to a stop and then immediately set off again after each 5 m sprint. Computer derived audible beeps were again used to “pace” the completion of the bouts of activity. Participants maintained ST sprinting until the volume of activity matched that completed at voluntary exhaustion in the COD trial. Therefore, the key parameters of intensity (speed and number of accelerations/decelerations) and volume (distance and total time of exercise) was matched between COD and ST trials. Therefore, the only difference between the trials was that in COD, the participants changed direction whereas in the ST trial they continued in the same direction.

Dependent variables associated with movement, physiological and subjective outcomes were recorded to comprehensively describe the exercise in COD and ST trials and the associated demand. Prior to the exercise, an athlete tracking unit that included a micro-electrical mechanical device and global positioning system (Catapult S5, Australia) was fitted into a purpose made vest and placed on each participant so it was positioned between the shoulder blades. All participants used the same tracking unit throughout the testing session to avoid inter-unit variability of the measurement (Nicolella et al., 2018). A short-range radio telemetry device to monitor heart rate was also fitted (Polar T31 Kempele, Finland) according to manufactures guidelines. Both movement and heart rate were recorded continuously throughout the exercise session. Following the completion of the experimental protocol, data was downloaded using manufacturers software (Catapult Openfield software pack) to obtain the peak heart rate observed in COD and ST trials and the peak Playerload^TM^ [the instantaneous rate of change of acceleration divided by a scaling factor, used as an indicator of the overall movement demand (Nicolella et al., 2018)]. During both experimental protocols between the 30 s rest periods between sprints, participants were asked to self-rate their differential RPE for both local (legs) and central (breathlessness) exertion using a Centimax 100 scale (Weston et al., 2015). This data provided a subjective evaluation of the central and peripheral demand associated with the trials. The peak differential RPE for both legs and breathlessness were used to compare subjective ratings between COD and ST. Capillary blood lactate was determined at rest (baseline) and post exercise from a fingertip blood sample for each participant. Following a puncture made using a lancet (Accu-Chek, Safe-T Pro Plus) a blood sample was drawn from the fingertip, using a 20 μl blood capillary tube. The blood sample was immediately placed and mixed into the lactate hemolyzing solution cup, to prevent the blood sample from clotting. All samples were analyzed using Biosen C-Line “EKF diagnostic” device. Pre-blood lactate sample was drawn at rest before the warm-up, and post-blood lactate was drawn 5 min following the completion of the exercise trial.

### Skeletal Muscle Biopsies

Muscle biopsies were taken from the lateral portion of the vastus lateralis muscle under local anesthetic with Marcain (0.5%) using the conchotome technique (100–150 mg tissue per biopsy) (Patel et al., 2011). Due to ethical considerations of multiple biopsies from the same leg over a short time period, within each trial muscle biopsies were taken from both legs (specific details below). Upon arrival at the laboratory under overnight fasted conditions, the pre-exercise muscle biopsy was collected from the non-dominant leg. Post-exercise muscle biopsies were taken (from the dominant leg) 30 min following the completion of COD and ST exercise, at 3 h (from the dominant leg using a separate incision at least 3 cm from the 30 min biopsy site) for gene expression analysis (described below). A final biopsy was taken 24-h post-exercise again following an overnight fast (from the non-dominant leg). Data collected in the development of this experimental protocol did not observe any significant differences between the muscle activation (as measured by EMG analysis) between the non-dominant and dominant legs in a similar COD protocol (0.57 ± 0.09 vs. 0.58 ± 0.13 μV, respectively). This would suggest that there would be limited differences between the response to exercise during such protocols irrespective of the potential dominance of a specific limb during COD movements. All biopsies were taken at a similar time of day (all AM, with all 3 h biopsies taken early in the PM) and all across the summer period.

### Tissue Homogenization, DNA Isolation, and Bisulfite Conversion

After all five subjects were used for physiological measures, power analysis was conducted to detect a greater than 1.05 (5%) fold change in methylation based on our previous studies (Seaborne et al., 2018a), *n* = 3 in a within-subject design was determined as sufficient to detect statistically significant changes in methylation over both trials and over the baseline, post and 24 h timepoints. Therefore, three subjects were chosen at random (i.e., not pre-selected or biased selection) out of the five well-trained male team sports players (age: 27 ± 2 years; height: 1.7 ± 0.1 m; weight: 73.5 ± 11.1 kg) for DNA methylome analysis at baseline, 30 min post and 24 h post in both COD and ST trials. Note that methylation EPIC bead chip data (described below) for baseline rested samples in these young adults has also been used in an epigenomic comparison with elderly rested muscle tissue in our recent publication (Turner et al., 2020). However, samples were originally derived from the present study and design described above, therefore are the relevant baseline samples for comparisons in the present study. Samples have been deposited in open access repository GEO: GSE162288. Tissue samples were homogenized for 45 s at 6,000 rpm × 3 (5 min on ice in between intervals) in lysis buffer (180 μl buffer ATL with 20 μl proteinase K) provided in the DNeasy spin column kit (Qiagen, United Kingdom) using a Roche Magnalyser instrument and homogenization tubes containing ceramic beads (Roche, United Kingdom). The DNA was then bisulfite converted using the EZ DNA Methylation Kit (Zymo Research, CA, United States) as per manufacturer’s instructions.

### Infinium Methylation EPIC BeadChip Array

All DNA methylation experiments were performed in accordance with Illumina manufacturer instructions for the Infinium Methylation EPIC BeadChip Array. Methods for the amplification, fragmentation, precipitation and resuspension of amplified DNA, hybridization to EPIC BeadChip, extension and staining of the bisulfite converted DNA (BCD) can be found in detail in our open access methods paper (Seaborne et al., 2018b). EPIC BeadChips were imaged using the Illumina iScan System (Illumina, United States).

### DNA Methylation Analysis, CpG Enrichment Analysis (GO and KEGG Pathways), Differentially Modified Region Analysis and Self Organizing Map (SOM) Profiling

Following DNA methylation quantification via Methylation EPIC BeadChip array, raw. IDAT files were processed using Partek Genomics Suite V.7 (Partek Inc., MO, United States) and annotated using the MethylationEPIC_v-1-0_B4 manifest file. We first checked the average detection *p*-values. The mean detection *p*-value for all samples was 0.000141, and the highest was 0.00024 (Supplementary Image 1), which is well below the recommended 0.01 in the Oshlack workflow (Maksimovic et al., 2016). We also examined the raw intensities/signals of the probes, that demonstrated an average median methylated and unmethylated signal of over 11.5 (11.74) and the difference between the average median methylated and average median unmethylated signal was 0.28, well below the recommended difference of less than 0.5 (Maksimovic et al., 2016). Upon import of the data into Partek Genomics Suite we filtered out probes located in known single nucleotide polymorphisms (SNPs) and any known cross-reactive probes using previously defined SNP and cross-reactive probe lists identified in earlier EPIC BeadChip 850K validation studies (Pidsley et al., 2016). Although the average detection *p*-value for each sample across all probes was on average very low (no higher than 0.00024) we also excluded any individual probes with a detection *p*-value that was above 0.01 as recommended previously (Maksimovic et al., 2016). Therefore, out of a total of 865,860 probes, removal of known SNPs, cross-reactive probes and those with a detection *p*-value above 0.01 resulted in a final list of 809,877 probes analyzed. Following this, background normalization was performed via functional normalization (with noob background correction), as previously described (Maksimovic et al., 2012). Following functional normalization, we also undertook quality control procedures via principle component analysis (PCA), density plots by lines as well as box and whisker plots of the normalized data for all samples (Supplementary Images 1b–d, respectively). We confirmed that no samples demonstrated large variation [variation defined as any sample above 2 standard deviations (SDs) – depicted by ellipsoids in the PCA plots (Supplementary Image 1b) and/or demonstrating any differential distribution to other samples, depicted in the signal frequency by lines plots (Supplementary Image 1c)]. Therefore, no outliers were detected in this sample set. We used participants baseline samples from one trial only for array analysis, as these were resting biopsies from the same participant under the same conditions before each trial. We also confirmed that baseline samples were not different between COD and ST trials by running a participant’s COD and ST trial baseline samples and demonstrating that the PCA showed very little variation in methylation across the methylome between the rested baseline samples taken before the COD trial and the rested baseline sample taken before the ST trial (Supplementary Image 1c- Samples highlighted with an arrow). Following normalization and quality control procedures, we undertook differentially methylated position (DMP) analysis by converting β-values to *M*-values [*M*-value = log_2_(β/(1 − ββ)], as *M*-values show distributions that are more statistically valid for the differential analysis of methylation levels (Du et al., 2010). We performed a two-way ANOVA for ‘Trial’ (ST vs. COD) × time (baseline, post, 24 h) with planned contrasts of: (1) COD Post vs. ST post, (2) COD 24 h vs. ST 24 h). We also explored the ANOVA main effects for both ‘Trial’ to investigate the effect of trial alone (effect of COD vs. ST across time), and ‘Time’ (baseline, post and 24 h) with planned contrasts (post vs. baseline, baseline vs. 24 h, post vs. 24 h) to investigate the effect of time alone (exercise effect alone over time in both COD and ST groups). Any differentially methylated CpG position (DMP) with a *P*-value of ≤0.01 was used as the statistical cut off for the discovery of DMPs. We then undertook CpG enrichment analysis on these differentially methylated CpG lists within gene ontology (GO) and KEGG pathways (Kanehisa and Goto, 2000; Kanehisa et al., 2016, 2017) using Partek Genomics Suite, Partek Pathway and Revigo (tree-maps for GO terms). Differentially methylated region (DMR) analysis, that identifies where several CpGs are consistently differentially methylated within a short chromosomal location/region, was undertaken using the Bioconductor package DMRcate (DOI: 10.18129/B9.bioc.DMRcate). Finally, in order to plot/visualize temporal changes in methylation across the time-course post exercise (0, 30 min, 24 h) in both trials (COD vs. ST) we implemented Self Organizing Map (SOM) profiling of the change in mean methylation within each condition using Partek Genomics Suite.

### Comparative/Integrative Methylome and Transcriptome Analysis

We overlapped the significant DMP data from the present study with significantly differentially expressed genes identified in the sprint transcriptome (Rundqvist et al., 2019) and also with recent meta-analysis that combines transcriptome data from 66 published data sets in the most comprehensive exercise transcriptome profiling to date (Pillon et al., 2020), as available on the associated web interface MetaMex.eu (Pillon et al., 2020). Venn diagram analysis was used to generate gene lists of overlap between the significant DMP methylome data in the present manuscript with the statistically significant transcriptome data defined in the original studies. We also used this approach to determine if the genes that were identified to be hypomethylated in the present study and upregulated in the sprint transcriptome (Rundqvist et al., 2019), were also upregulated in the aerobic exercise transcriptome meta-analyses (Pillon et al., 2020), as well as genes that were hypermethylated in the present study and down-regulated in the transcriptome data sets. Meta-analyses data base, MetaMex (Pillon et al., 2020), was used to confirm the genes log fold change and significance (with an FDR of *p* ≤ 0.05) in the transcriptome data. In particular, for these transcriptome meta-analysis data we used the criteria of healthy skeletal muscle up to 24 h post exercise in order to more accurately match the samples obtained in the present study. Enabling us to remove non-equivalent time points or participants, e.g., diseased. To generate a list of genes that were significantly differentially methylated in the present study and significantly regulated at the expression level in transcriptome meta-analysis after acute aerobic exercise (but not identified in the sprint transcriptome) (Rundqvist et al., 2019) we conducted new analysis of acute aerobic exercise transcriptome datasets from the meta-analysis (Pillon et al., 2020). Therefore, we also analyzed significantly differentially expressed genes lists (adj. *p*-value of ≤0.05) that were up- or down-regulated in transcriptome meta-analysis after acute aerobic exercise (Pillon et al., 2020) and overlapped these via Venn-diagram analysis lists with our most significant methylated DMP analysis. New correlation analysis was performed on the acute aerobic meta-analysis data (Pillon et al., 2020) for the genes identified in the present manuscript at the methylome, comparative transcriptome and targeted gene level (RT-PCR below) to be significantly altered (including *VEGFA*, *NR4A1, NR4A3* and *PPARGC1A*).

### RNA Isolation, Primer Design, and Gene Expression Analysis

Skeletal muscle tissue muscle from the same samples as the DNA methylome analysis across both trials (ST and COD) at baseline (pre exercise), 30 min and 24 h post, as well as an additional biopsy at 3 h was used for targeted gene expression analysis. This is because ourselves and other have previously demonstrated large changes in methylation after exercise at earlier timepoints, e.g., 30 min (Seaborne et al., 2018a, b) preceding significant changes in gene expression that significantly increases later (e.g., between 3 and 6 h post exercise). Muscle was homogenized in tubes containing ceramic beads (MagNA Lyser Green Beads, Roche, Germany) and 1 ml Tri-Reagent (Invitrogen, Loughborough, United Kingdom) for 45 s at 6,000 rpm × 3 (and placed on ice for 5 min at the end of each 45 s homogenization) using a Roche Magnalyser instrument (Roche, Germany). RNA was then isolated as per Invitrogen’s manufacturer’s instructions for Tri-reagent. Then a one-step RT-PCR reaction (reverse transcription and PCR) was performed using QuantiFast SYBR Green RT-PCR one-step kits on a Rotorgene 3000Q. Each reaction was setup as follows; 4.75 μl experimental sample (7.36 ng/μl totaling 35 ng per reaction), 0.075 μl of both forward and reverse primer of the gene of interest (100 μM stock suspension), 0.1 μl of QuantiFast RT Mix (Qiagen, Manchester, United Kingdom) and 5 μl of QuantiFast SYBR Green RT-PCR Master Mix (Qiagen, Manchester, United Kingdom). Reverse transcription was initiated with a hold at 50°C for 10 min (cDNA synthesis) and a 5 min hold at 95°C (transcriptase inactivation and initial denaturation), before 40–50 PCR cycles of; 95°C for 10 s (denaturation) followed by 60°C for 30 s (annealing and extension). Primer sequences for genes of interest and reference genes were. *VEGFA* Fwd ACGGTCCCTCTTGGAATTGG, Rvse CTAATCT TCCGGGCTCGGTG; *NR4A1* Fwd GGTGACCCCACGATTT GTCT, Rvse GGCTTATTTACAGCACGGCG; *NR4A3* Fwd GACGTCGAAACCGATGTCAG, Rvse TTTGGAAGGCAGA CGACCTC, *PPARGC1A* Fwd TGCTAAACGACTCCGAGAA, Rvse TGCAAAGTTCCCTCTCTGCT; *RPL13A* Fwd GGCTA AACAGGTACTGCTGGG, Rvse AGGAAAGCCAGGTACTTC AACTT. All primers were designed to yield products that included the majority of transcript variants for each gene as an impression of total changes in the gene of interests’ expression levels. All genes demonstrated no unintended gene targets via BLAST search and yielded a single peak after melt curve analysis conducted after the PCR step above. All relative gene expression was quantified using the comparative Ct (^ΔΔ^Ct) method (Schmittgen and Livak, 2008). The baseline sample for each participant and their own reference gene sample was used as the calibrator conditions. The average, standard deviation and variations in Ct value for the *RPL13A* reference gene demonstrated low variation across all samples (mean ± SD, 21.04 ± 1.49, 7.1% variation) for the analysis. The average PCR efficiencies for *VEGFA, NR4A1, NR4A3* and *PPARGC1A* were comparable (90 ± 5.2, 89.2 ± 4.2, 91.3 ± 6.4%, 92.5 ± 1.9 variation) with the reference gene *RPL13A* (89.1 ± 4.8%). Statistical analysis genes were performed on *n* = 3 in duplicate using a two-way ANOVA with fisher *post hoc* comparisons at the level of *P*-value of ≤0.05 using Graphpad.

## Results

### Change of Direction Exercise Elicits Increased Loading Compared With ST Exercise

Change of direction exercise had significantly higher mean movement loading (GPS Playerload) (3.35 ± 0.07 vs. 2.93 ± 0.08 au; *p* ≤ 0.001), physiological loading (heart rate post exercise) (159 ± 16.3 vs. 137 ± 13.5 bpm; *p* ≤ 0.001), metabolic loading (lactate 5 min post exercise) (8.45 ± 1.7 vs. 1.69 ± 0.68 mmol/L; *p* ≤ 0.05), subjective (differential RPE) peripheral loading (47 ± 24 vs. 10 ± 6; *p* ≤ 0.001) and central loading (62 ± 29 vs. 10 ± 6; *p* ≤ 0.001) versus ST exercise.

### Exercise Alone Evokes a Hypermethylated Response 30 Min Post Exercise Followed by Hypomethylated Signature by 24 h, Particularly in MAPK, AMPK and Axon Guidance Pathways

**FIGURE 1:**
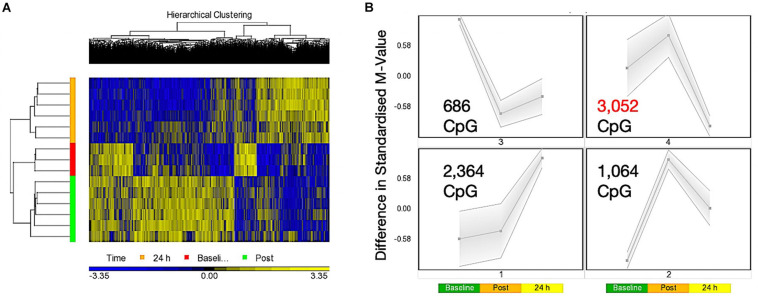
**(A)** Hierarchical heat map for analysis of DMPs over exercise time in both trials combined (Red – Baseline, Post = 30 min post – Green, 24 h – Orange). **(B)** SOM temporal analysis of DMPs over exercise time in both trials. Post = 30 min post exercise.

A one-way ANOVA for ‘time’ (baseline, 30 min post, 24 h post exercise), that analyses the main effect of exercise alone over time across both experimental trials combined (COD and ST trials), identified a list of 7,612 significantly differentially methylated CpG positions (DMP’s) (Supplementary Table 1a). Within this ‘exercise alone’ list, 1,076 DMPs were promoter associated with 1,651 located in CpG islands, with the majority (1,253) demonstrating hypermethylation. Out of the 1,651 located in CpG islands 735 DMPs were also located in promoters, with seven of these DMPS also located in Phantom5 enhancer regions. The remaining DMPs were located in N-Shelf (273), N_Shore (712), S-Shelf (259), S_Shore (603), with 4,114 characterized as unclassified. Planned contrasts within this ANOVA for baseline vs. post exercise time points, also identified 4,952 significant DMPs (Supplementary Table 1b), with a larger number of DMPs hypermethylated post exercise versus baseline (3287 hyper- and 1665 hypo-methylated). At 24 h (24 h vs. baseline contrast) there was 6,638 DMPs (Supplementary Table 1c), that demonstrated a shift toward a hypomethylated profile at 24 h, with 3,617 hypermethylated vs. 3,021 hypomethylated DMPs. Furthermore, this shift was confirmed in the 24 h vs. 30 min post contrast, that identified a DMP list of 13,139 CpGs (Supplementary Table 1d), with a predominately hypomethylated profile of 8,413 hypomethylated vs. 4726 hypermethylated DMPs. These data therefore suggest that 30 min post exercise (across both experimental trials, COD and ST) DMPs were predominantly hypermethylated, however, after that point and up to 24 h there was shift toward a hypomethylated profile. This observation can be visualized as a hierarchical heat map in Figure 1A. Finally, SOM temporal analysis of the 7,612 DMPs also confirmed the above analysis and suggested that the largest group of DMPs (3,052 CpG’s highlighted in red and located in SOM profile; Figure 1B) were hypermethylated 30 min post exercise, with the same DMPs demonstrating a shift to a hypomethylated profile 24 h post exercise.

We next wished to identify which gene ontology (GO) terms and KEGG pathways were significantly enriched over the time-course of baseline, 30 min post and 24 following exercise (across both experimental trials (COD and ST). Indeed, GO enrichment analysis of the post vs. baseline contrast (Supplementary Table 1e) identified that there was significant hypermethylation enrichment of DMPs in top five GO terms: (1) Neuron Part, (2) developmental process, (3) cellular component organization, (4) regulation of neuron projection development and, (5) cellular component organization or biogenesis (Supplementary Tables 1f–j, respectively). For the 24 h versus baseline contrast (Supplementary Table 1k) DMP hyper/hypomethylation was more balanced in top five GO terms: (1) Protein binding, (2) binding, (3) organelle, (4) intracellular part, and (5) intracellular organelle (Supplementary Tables 1l–p). Then at 24 h compared with 30 min post exercise (Supplementary Table 1q) top five GO terms demonstrating enriched hypomethylation were: (1) Developmental process, (2) anatomical structure development, (3) anatomical structure morphogenesis, (4) regulation of signaling and, (5) regulation of cell communication (Supplementary Tables 1r–v).

For KEGG pathway analysis, in post vs. baseline contrasts (Supplementary Table 2a) there was significant enrichment in hypermethylation of DMPs in top five KEGG pathways including: (1) MAPK signaling pathway, (2) axon guidance, (3) human papillomavirus infection, (4) small cell lung cancer and, (5) insulin secretion (Supplementary Tables 2b–f). At 24 h vs. baseline (Supplementary Table 2g) top five enriched KEGG pathways were: (1) MAPK signaling pathway, (2) AMPK signaling pathway, (3) lysine degradation, (4) phosphonate and phosphate metabolism and, (5) aldosterone synthesis and secretion (Supplementary Tables 2h–l). At 24 h vs. post exercise (Supplementary Table 2m) demonstrating a hypomethylated profile were enriched in top five KEGG pathways: (1) Axon guidance, (2) cGMP-PKG signaling pathway, (3) focal adhesion, (4) rap1 signaling, and (5) adrenergic signaling in cardiomyocytes’ (Supplementary Tables 2n–r).

Finally, DMR analysis of the 30 min post vs. baseline (Supplementary Table 2s), 24 h vs. baseline (Supplementary Table 2t) and 24 h vs. 30 min post contrasts (Supplementary Table 2u) identified several regions located in or close to annotated genes with enriched (multiple CpGs in short chromosomal regions) differential methylation. Including enriched hypermethylation of genes: *RBMXL1*, *ALDH3A1*, *EVA1A*, *MiR3928*, *RNF185*, *ANAPC10*, and *ABCE1* (30 min post vs. baseline) and *c12orf42*, *HSPD1*, *HSPE1*, *SALL1*, *CCNDN2* (24 h vs. baseline) and enriched hypomethylation in genes: *ZIC1*, *ZIC4*, *TBX15*, *NAV2*, and *OXT* (24 h vs. 30 min post).

### Change of Direction Exercise Evokes a Greater Hypomethylated Signature in Protein Binding and Axon Guidance Pathways Compared With Straight Line Exercise

**FIGURE 2:**
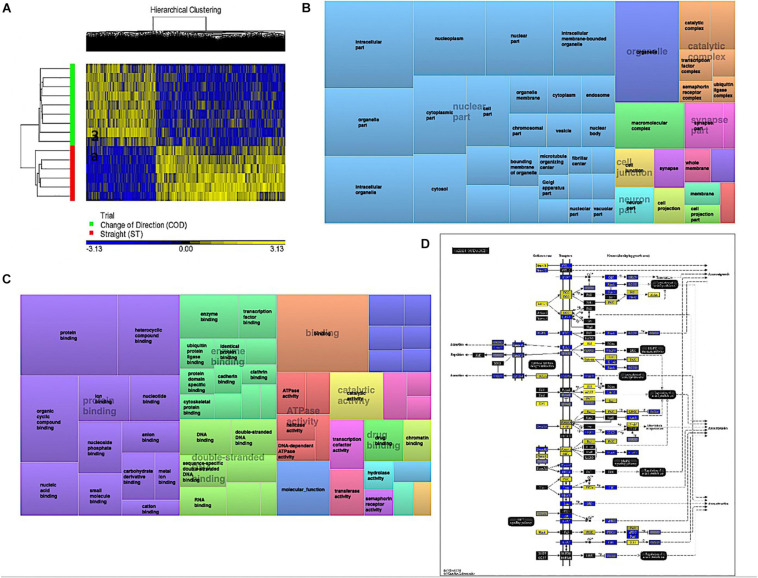
**(A)** Hierarchical heat map for analysis of DMPs in COD vs. ST trials across all time. **(B)** DMP enrichment TreeMap of overarching Gene Ontologies (GO) term ‘cellular component’ in COD vs. ST trials. Demonstrating top enriched GO terms of ‘intracellular part,’ ‘organelle,’ and ‘intracellular organelle part.’ **(C)** DMP enrichment TreeMap of overarching GO ‘molecular function.’ Demonstrating top enriched GO terms of ‘protein binding’ and ‘binding.’ **(D)** Illustration of the top KEGG pathway ‘axon guidance’ demonstrating predominantly hypomethylation (blue) in COD vs. ST sprint trials. Note, the most significant (lowest *p*-value) DMP for each gene is used to color this pathway image. Therefore, this is not always accurate where multiple DMPs occur for a single gene and the image is therefore only a visual representation of the overarching methylation profile in this pathway. Full and accurate DMP lists for the axon guidance pathway in these conditions can be found in Supplementary Table 3i. Figure adapted with permission from Kanehisa and Goto (2000) and Kanehisa et al. (2016, 2017).

Analyzing the main effect for ‘Trial’ (COD vs. ST sprint exercise across all time points) identified 13,218 significant DMPs (Supplementary Table 3a) favoring hypomethylation over hypermethylation (8,627 hypo- vs. 4,654 hyper-methylated). Undertaking hierarchical clustering of these DMPs enabled visualization of the predominance in hypomethylation in COD versus the ST exercise trials (Figure 2A). A large number of DMPs 4,221 (out of 13,218) were promoter associated, with 5,304 (out of 13,218) located in CpG islands, and 2,969 located in both CpG island and promoters. Undertaking gene ontology (GO) pathway enrichment within this 13,218 DMP list (Supplementary Table 3b), the top five GO terms enriched for hypomethylation in COD vs. ST sprint exercise were: (1) Intracellular part, (2) protein binding, (3) binding, (4) organelle and, (5) intracellular organelle part (Supplementary Tables 3c–g), predominantly within overarching ‘cellular component’ (Figure 2B) and ‘molecular function’ gene ontologies (Figure 2C). The top KEGG pathways (Supplementary Table 3h) predominantly hypomethylated were: (1) Axon guidance (Figure 2D), (2) cell cycle, (3) endocytosis, (4) pathways in cancer and, (5) spliceosome (Supplementary Tables 3i–m, respectively). DMR analysis also identified that genes: *E2F3, BRD2, MUS81* and *CFL1* had enriched hypomethylation of multiple DMPs in short chromosomal regions in COD versus ST sprint exercise (Supplementary Table 3n).

### Change of Direction Exercise Evokes the Largest Hypomethylated Signature 30 Min Post Exercise Compared With Straight Line Exercise, Particularly in Protein Binding, Axon Guidance and Insulin Related Pathways

**FIGURE 3:**
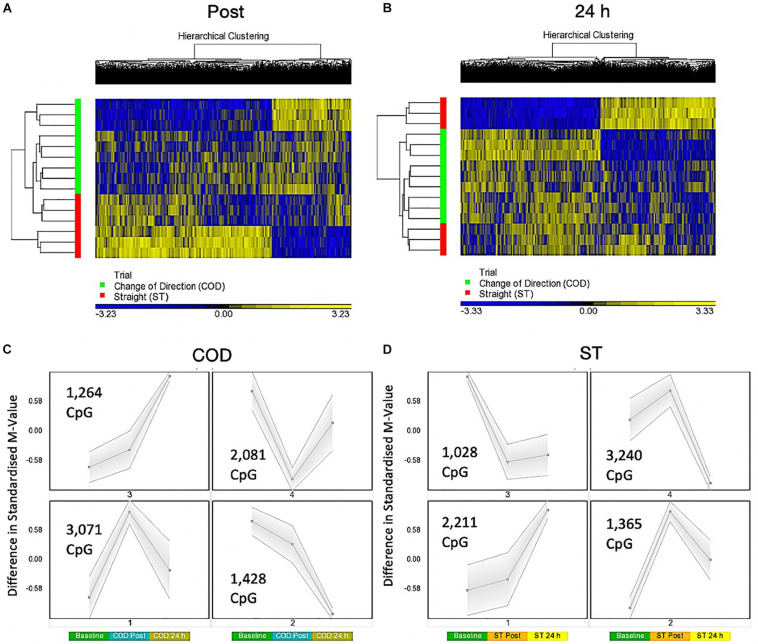
**(A)** Hierarchical heat map for analysis of DMPs in COD vs. ST trials 30 min (post) exercise. **(B)** Hierarchical heat map for analysis of DMPs in COD vs. ST trials 24 h post exercise. **(C)** SOM temporal analysis of DMPs over exercise time in COD exercise trial at baseline, post and 24 h post exercise. **(D)** SOM temporal analysis of DMPs over exercise time in ST exercise trial at baseline, post and 24 h post exercise.

A two-way ANOVA for ‘Trial’ × ‘Time’ interaction, that demonstrates a difference in methylation between COD and ST sprint exercise 30 min post and 24 h post exercise, identified 7,844 significant DMPs (Supplementary Table 4a). 1,349 DMPs were promoter associated with 1,846 DMPs within CpG islands and 883 located in both promoters and islands. Contrasts for COD 30 min post vs. ST post identified 6,489 significant DMPs (Supplementary Table 4b), where hypomethylation was predominant post exercise in COD vs. ST post exercise (4,472 hypomethylated and 2,017 hypermethylated DMPs) (Figure 3A). At 24 h (COD 24 h vs. ST 24 h) there were 6,647 significant DMPs (Supplementary Table 4c), where the shift in favoring hypomethylation post exercise was more balanced with hypermethylation at 24 h, yet still with a larger total of hypomethylated (3,649) compared with hypermethylated (2,953) DMPs (Figure 3B). Furthermore, SOM analysis of mean temporal changes generated over time from the mean of each condition using the interaction Trial × Time (7,844 DMP) list demonstrated the following temporal profiles depicted in Figure 3C for COD exercise and Figure 3D for ST exercise. These profiles confirmed that the majority of DMPs in the COD trial demonstrated a hypomethylated compared with hypermethylated profile particularly at the 30 min post exercise timepoint.

In gene ontology (GO) analysis of the two-way ANOVA ‘Trial’ × ‘Time’ interaction (7,844 DMP list; Supplementary Table 4d), identified overarching GO terms: Biological process, cellular component, and molecular function, as well as the top GO term protein binding (within molecular function) (Supplementary Table 4e) that demonstrated more DMPs hypomethylated compared with hypermethylated (2,276 hypomethylated versus 1,386 hypermethylated). The same trend was observed after KEGG pathway analysis (Supplementary Table 4f) where the top differentially enriched pathway was ‘axon guidance’ (Supplementary Table 4g) with more DMPs hypomethylated in COD post vs. ST post exercise (61 hypomethylated, 44 hypermethylated).

More specifically 30 min post exercise in COD vs. ST trials, gene ontology (GO) analysis of the differentially methylated CpG’s within this contrast (6,489 DMP list above; Supplementary Table 4h) demonstrated the most significantly enriched top five GO terms as: (1) Protein binding (Supplementary Table 4i), (2) binding, (3) positive regulation of cellular metabolic process, (4) positive regulation of metabolic process, and (5) positive regulation of macromolecule metabolic process (Supplementary Tables 4j–m) that all demonstrated a larger number of DMPs that were hypomethylated vs. hypermethylated in these GO terms. By 24 h in the 6,647 DMP list (contrast COD 24 h vs. ST 24 h) there was a shift back to a more balanced hypo/hypermethylated ratio in the top five GO terms (Supplementary Table 4n) for: (1) protein binding (Supplementary Table 4o), (2) binding, (3) regulation of signaling, (4) regulation of cell communication, and (5) anatomical structure morphogenesis; Supplementary Tables 4p–s).

The same trend was observed after KEGG pathway analysis (Supplementary Table 4t) where the top differentially enriched hypomethylated pathways were: (1) insulin resistance, (2) endocytosis, (3) axon guidance, (4) MAPK signaling pathway and, (5) insulin signaling pathway in the COD 30 min post vs. ST 30 min post contrast (Supplementary Tables 4u–y). In the 24 h vs. 24 h ST trial contrast KEGG pathway analysis (Supplementary Table 5a) with more balanced hypo/hypermethylation ratio: (1) ErbB signaling, (2) Non-small cell lung cancer, (3) axon guidance, (4) proteoglycans in cancer, and (5) glioma (Supplementary Tables 5b–f). In DMR analysis COD 30 min post vs. ST 30 min post (Supplementary Table 5g), identified enriched hypomethylation in genes: *CIITA, PRR5, WDR46/PFDN6*, *MAGI2, RNF167*, and *DGKZ*. In COD 24 h vs. ST 24 h DMR analysis identified genes *COL1A1* and *CHID1* possessed enriched hypermethylation (Supplementary Table 5h).

### Comparative Methylome and Transcriptome Analyses Identifies Inverse Relationships in DNA Methylation and Gene Expression: NR4A1 Is Hypomethylated and Associated With Increased Gene Expression After Acute Exercise

**FIGURE 4:**
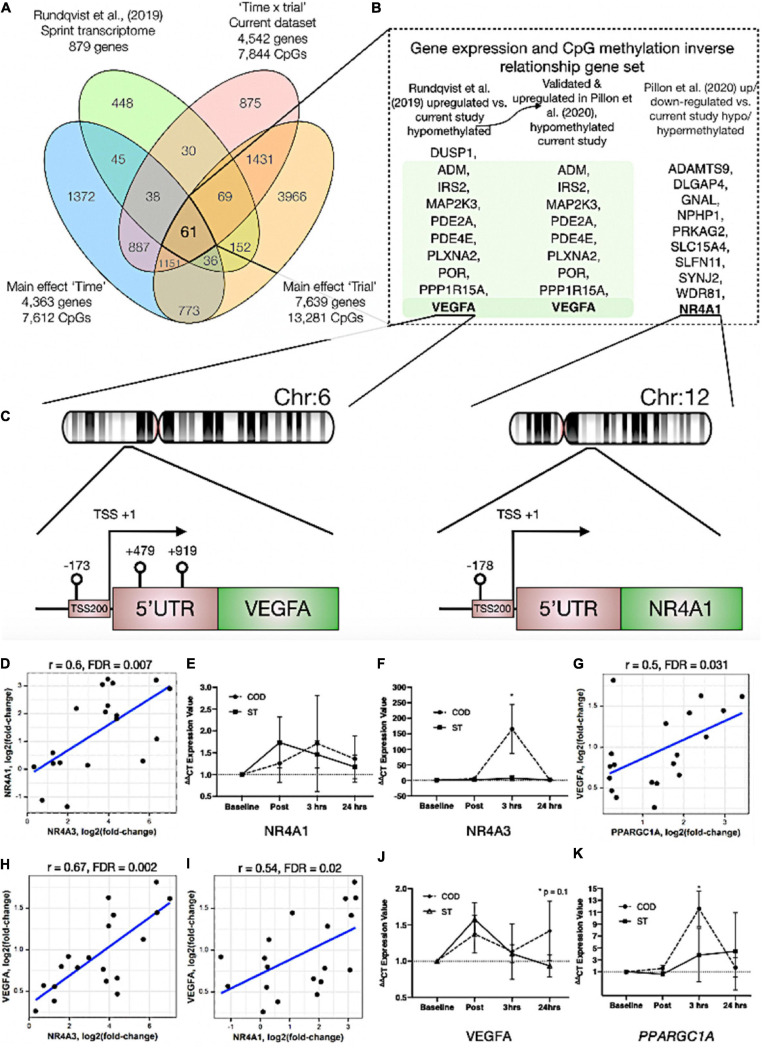
**(A)** Venn diagram analyses of the overlap between the sprint transcriptome (Rundqvist et al., 2019) and differential methylome analysis in the present study. A total of 431 genes overlapped with the sprint transcriptome and were identified as significant DMPs in at least one of the methylome analyses in the present study. Sixty one genes were and up/down regulated in the sprint transcriptome and significantly differentially methylated across all methylome analyses in the present study. **(B)** Genes (shaded in green) out of the 61 identified in **(A)**, that were significantly upregulated in the sprint transcriptome (Rundqvist et al., 2019), upregulated in acute aerobic exercise transcriptome meta-analysis (Pillon et al., 2020) as well as hypomethylated in all methylome analyses in the present study. Unshaded genes were upregulated in acute aerobic exercise transcriptome meta-analysis (Pillon et al., 2020) (but not changed in the sprint transcriptome) and significantly hypo or hyper methylated in all methylome analyses in the present study. **(C)** Demonstrates a schematic of the hypomethylated chromosomal regions close to the transcription start sites for gene VEGFA (left) and NR4A1 (right) with change of direction exercise. **(D)** Highly significant positive correlations in gene expression for *NR4A1 vs. NR4A3.* **(E)** Gene expression of *NR4A1* and **(F)** *NR4A3* at post, 3 h and 24 h after COD vs. ST exercise. Highly significant positive correlations in gene expression for **(G)** *VEGFA vs. PPARGC1A* (encodes PGC1-α), **(H)** *VEGFA vs. NR4A3* and **(I)** *VEGFA vs. NR4A1* in young healthy adult skeletal muscle 30 min post up to 24 h post exercise in new reanalysis of all acute aerobic transcriptomes meta-analyses (Pillon et al., 2020). Gene expression for **(J)** *VEGFA* and **(K)** *PPARGC1A* at post, 3 h and 24 h after COD vs. ST exercise. *p ≤ 0.05 unless otherwise stated.

In order to identify genes that were differentially methylated and also altered at the gene expression level after exercise between COD and ST exercise trials, we first overlapped the present studies methylome data (significant DMPs main effect for ‘time’ 7,612 DMP list, main effect for ‘trial’ 13,281 DMP list and the interaction ‘time’ × ‘trial’ 7,844 DMP list) with recent published transcriptome data sets following acute sprint exercise (Rundqvist et al., 2019). In this sprint transcriptome, the authors identified 879 genes that were significantly differentially expressed post-exercise (471 upregulated/408 downregulated - List in Supplementary Table 6a). Out of these 879 genes that had altered gene expression across the sprint transcriptome, there was large overlap of genes (49% or 431 genes) that were also differentially methylated in at least one of the significant DMP analyses in the present study (Figure 4A and Supplementary Table 6b for gene list). To determine genes that had frequently occurring DMPs in COD vs. ST sprint exercise trials and that mapped through to significant differential gene expression in the transcriptome analysis, using Venn diagrams we first identified that out of these 431 genes, 61 were shared by all methylation analyses in the present study (Figure 4Aa and Supplementary Table 6c). Eighteen of the genes that were upregulated, also displayed hypomethylation (on 27 DMPs) 30 min post exercise in COD vs. ST exercise conditions (Supplementary Table 6d). Fifteen of the genes, that were downregulated were also hypermethylated (24 DMPs) 30 min post exercise in COD vs. ST conditions (Supplementary Table 6e). Fifteen genes were also upregulated and hypomethylated (21 DMPs) 24 h post exercise (Supplementary Table 6f) and twenty-one of these genes were downregulated and hypermethylated (30 DMPs) 24 h post exercise (Supplementary Table 6g). Those genes with the same trend (down and hypermethylated) at both time points (30 min post and 24 h) in COD vs. ST exercise trials, included a final list of 13 genes (containing 28 DMPs) including: *ATP2C1, CCDC88C, FAM111A, GAB1, HMCN1, LRRTM4, LTBP1, MAGI1, MYH10, MYO10, NEDD9, NIPAL3* and *RHOBTB1* (Supplementary Table 6h). With 10 genes (containing 22 DMPs) that were upregulated and hypomethylated at both 30 min post and 24 h in COD vs. ST sprint exercise trials, including genes: *ADM, DUSP1, IRS2, MAP2K3, PDE2A, PDE4E, PLXNA2, POR, PPP1R15A* and *VEGFA* (depicted Figure 4B and Supplementary Table 6i). The majority of these hypomethylated and upregulated genes were associated with the MAPK pathway, together with the canonical angiogenesis gene, *VEGFA* (see additional DMR data for *VEGFA* below).

In addition, we looked to validate if the genes identified in the present study’s methylome and recent sprint transcriptome were also regulated in the most comprehensive set of acute aerobic transcriptomes to date (Pillon et al., 2020). Indeed, the vast majority of the 13 genes found to be hypermethylated/down-regulated and 10 genes hypomethylated/up-regulated in the present study’s methylome and sprint transcriptome (Rundqvist et al., 2019), were all significantly regulated (FDR < 0.05; Metamex.eu) at the gene expression level (except two genes; *DUSP1* and *NEDD9*) in healthy skeletal muscle up to 24 h after acute aerobic exercise in the transcriptome meta-analysis (Pillon et al., 2020). Most importantly, in the meta-analyses’ acute aerobic transcriptome data all these genes were regulated in same direction as identified above. This comparative methylome and transcriptome analysis therefore determined that there was an inverse relationship between these genes’ DNA methylation profile and their gene expression after acute exercise. Furthermore, re-analyzing the full gene lists in the acute aerobic exercise transcriptomes meta-analysis data set (Pillon et al., 2020), we identified an additional 10 genes that were significantly up/down-regulated in the transcriptome meta-analyses (but not identified in the sprint transcriptome), that also demonstrated significantly altered methylation across all analyses in the present study (Figure 4A). These included: *NR4A1, ADAMTS9, DLGAP4, GNAL, NPHP1, PRKAG2, SLC15A4, SLFN11, SYNJ2, WDR81* (Figure 4B and Supplementary Tables 7a,b). Notably, within this list, *NR4A1* is a nuclear receptor and family member to *NR4A3* that has been identified as one of the most exercise and inactivity responsive genes across all 66 published transcriptome data sets (Pillon et al., 2020). *NR4A3* has a role in mediating the metabolic responses to exercise-like stimuli *in vitro* (Pillon et al., 2020). *NR4A1* is also within KEGG pathway of ‘MAPK signaling’ and GO term ‘protein binding’ identified above as enriched hypomethylated pathways in COD vs. ST exercise. Here, we identify that *NR4A1*, was hypomethylated in its promoter located in a CpG island close to its transcription start site TSS200 (cg11666140) in COD vs. ST exercise trials (Figure 4C (right image) and Supplementary Table 7a), with another s-shelf associated CpG also identified (cg20661548) to be hypomethylated 30 min post exercise in COD vs. ST trials (Supplementary Table 7b). Further analysis shows that, alongside NR4A3, NR4A1 gene expression was also significantly upregulated (0.94 log fold) in exercise-transcriptome meta-analyses (Pillon et al., 2020) and within the top ten genes upregulated in the sprint transcriptome (Rundqvist et al., 2019). *NR4A1* expression was also ranked second highest in its significant positive correlation with *NR4A3* [*r* = 0.72, metamex.eu (Pillon et al., 2020)] across the 66 published exercise and inactivity transcriptome data sets [metamex.eu (Pillon et al., 2020)], with new analysis of *NR4A1 and NR4A3* genes within meta-analyses transcriptomes of healthy young adults up to 24 h (to match the present studies samples) also suggesting a highly significant positive correlation (*r* = 0.6, FDR = 0.007; Figure 4D). Indeed, we were able to further observe an elevation in *NR4A1* gene expression at 3 h and 24 h post exercise in COD vs. ST trials, but this was not statistically significant (Figure 4E). Importantly however, we observed significant increases (*p* ≤ 0.05) of a large magnitude in *NR4A3* gene expression (165.2 vs. 6.8-fold, *p* ≤ 0.05) in COD vs. ST trial at 3 h post exercise (Figure 4F). Therefore, the comparative analysis of our present methylome data and its overlap across recent sprint transcriptome (Rundqvist et al., 2019) and meta-analysis transcriptomes after acute aerobic exercise (Pillon et al., 2020), provides evidence to suggest that there is an inverse relationship between the above genes methylation and their gene expression profiles. Also identifying for the first time, that *NR4A1* is an epigenetically regulated exercise gene and that *NR4A3* is upregulated to a large magnitude (165-fold) as a consequence of performing high intensity COD compared with ST sprint exercise.

### VEGFA Has Enriched Hypomethylation in Its Promoter and Is Strongly Associated With Increased Gene Expression Across Exercise Transcriptomes

We identified above that VEGFA was hypomethylated in the present study and gene expression increased across sprint and acute aerobic exercise meta-analysis transcriptomes. DMR analysis also identified enrichment of hypomethylation (3 DMPs) on the *VEGFA* gene (DMPs- cg01116220, cg04629501 and cg21099624 – Figure 4C (left image) and Supplementary Table 8a) in COD vs. ST exercise trails. All these DMPs were located amongst a CpG island within a 1,093 base pair locus. Two of these DMPs were also within *VEGFA*s promoter region close to its transcription start site (TSS) and first exon (cg21099624 - TSS200, cg01116220 - Exon 1, Figure 4C (left image) and Supplementary Table 8b). Overall, suggesting that this gene has enriched hypomethylation in its promoter in COD compared with ST sprint exercise trials. Finally, as mentioned above, *VEGFA* expression was significantly increased in the sprint transcriptome data set (Rundqvist et al., 2019), but was also one of the most upregulated genes (0.96 log fold change; metamex.eu) in the transcriptome meta-analysis for acute aerobic exercise in healthy males up to 24 h after exercise (Pillon et al., 2020). *VEGFA* was also ranked top (first) most significantly correlated gene at the expression level with the canonical exercise/metabolic regulators *PPARGC1A* (*r* = 0.84, metamex.eu) and *NR4A3* [*r* = 0.74, metamex.eu (Pillon et al., 2020)], that was the most significantly upregulated across all modes of exercise (Pillon et al., 2020) and identified as hypomethylated in its promoter in the present study. Correlation analysis of *VEGFA* with *PPARGC1A* and *NR4A3* genes within meta-analyses transcriptomes of healthy young adults 30 min post and up to 24 h post-acute aerobic exercise also suggested a significant correlation between *VEGFA and PPARGC1A* (*r* = 0.5, FDR = 0.031; Figure 4G) and *NR4A3* (*r* = 0.67, FDR = 0.002; Figure 4H). Further, *VEGFA* was significantly positively correlated with *NR4A1* [*r* = 0.58, metamex.eu (Pillon et al., 2020)] across all exercise transcriptomes and also after analysis of acute-aerobic exercise transcriptomes in healthy young adults (*r* = 0.54, FDR = 0.02; Figure 4I). Importantly, we identified *VEGFA* to possess enriched hypomethylation in its promoter in the current study. New interrogation of aged skeletal muscle tissue methylome in our recent studies, also suggests one of the same *VEGFA* CpG sites (cg04629501) is oppositely regulated (hypermethylated) with age (Turner et al., 2020) compared with exercise (hypomethylated) in the present study. Therefore, *VEGFA* is hypomethylated in its promoter and is associated with its own increased gene expression (as well as highly correlated with *PPARGC1A, NR4A1* and *NR4A3* expression) in the post-acute aerobic exercise transcriptome. Finally, *VEGFA* gene expression was elevated at 24 h (*p* = 0.01) in COD vs. ST trials (Figure 4J). We also observed significant increases (*p* ≤ 0.05) of a large magnitude in canonical metabolic gene, *PPARGC1A* (11.6 vs. 3.8-fold in COD vs. ST) in COD vs. ST trial at 3 h post exercise (Figure 4K). Overall, for the first time this study identifies *VEGFA* as an epigenetically regulated gene in the response to acute exercise.

## Discussion

The present study aimed to investigate the methylome and transcriptome 30 min post and 24 h after acute exercise in two sport and exercise relevant sprint shuttle running protocols: (1) COD and, (2) ST running exercise. In line with our original hypothesis, we first demonstrated that while exercise trials were matched for classically defined intensity measures (speed × distance) and the number of acceleration and deceleration movements, by including changes of direction into the protocol elicited greater movement (GPS Playerload), physiological (HR), metabolic (lactate), subjective central and peripheral (differential RPE) loading compared with ST running. Therefore, the comparison of COD versus ST sprint exercise represented a suitable model to investigate the time-course of the comparative DNA methylome and transcriptome following increased exercise loading in human skeletal muscle using physiologically relevant movements predominant within many sporting/exercise contexts and health prescription settings. Also, in line with our original hypothesis, we identified that both sprint exercise conditions evoked extensive alterations in the methylome 30 min post and 24 h after exercise, particularly in MAPK, AMPK and axon guidance pathways. COD exercise evoked a greater hypomethylation response across the genome particularly enriched in: Protein binding, MAPK, AMPK, insulin and axon guidance pathways, specifically at 30 min post exercise, compared with ST exercise. Further, comparative transcriptome analysis with recent sprint running transcriptomes identified a considerable 49% overlap of genes altered at the expression level that were also differentially methylated after COD exercise. In particular, after differential methylated region (DMR) analysis of genes altered across the methylome, we identified that vascular endothelial growth factor A (*VEGFA*) and downstream nuclear transcription factor, *NR4A1*, possessed hypomethylation within their promoter regions. *VEGFA* and *NR4A1* were also significantly upregulated in both sprint transcriptomes and recent meta-analysis of 66 published exercise transcriptomes (Pillon et al., 2020). Furthermore, within these published meta-analyses, *VEGFA* was the most highly ranked, significantly and positively correlated gene with the expression of well-established metabolic regulators; PPARGC1A (that encodes *PCG1-*α) and *NR4A3* in human skeletal muscle after exercise (Pillon et al., 2020). In re-analyses of the transcriptomes meta-analyses to correspond more closely with our study design (acute exercise, 30 min up to 24 h post exercise in healthy skeletal muscle), we also identified significantly positive correlations between *VEGFA*, *PCG1-*α and *NR4A3* gene expression. In the present study we also confirmed increased gene expression of *VEGFA* at 24 h, and *PPARGC1A* and *NR4A3* at 3 h post COD vs. ST exercise. In summary, we demonstrated that increased physiological load via COD running exercise in human skeletal muscle evoked considerable epigenetic modifications that were associated with changes in expression of genes responsible for adaptation to exercise. Further, an implication of these findings may therefore suggest, by incorporating changes of direction into exercise regimes overtime may ultimately help improve performance within a sporting context or health outcomes in populations with dysfunction in these gene pathways (e.g. MAPK, insulin signaling).

Acute COD exercise evoked enriched hypomethylation in pathways such as insulin resistance and insulin signaling (specifically 30 min post exercise) that have been demonstrated to be hypomethylated in skeletal muscle of people with type-II diabetes following 6 months of aerobic exercise training (3 days/week) (Nitert et al., 2012), and in people with type-II diabetes that are non-responders to exercise (Stephens et al., 2018). This supports the notion that this type of change in direction exercise maybe beneficial for those with metabolic disease, providing this type of higher intensity exercise is tolerable for these individuals. Others have suggested that higher intensity exercise is well tolerated and also extremely practical, given that it provides a reduction in the time-burden of the exercise regime (Liu et al., 2019). Furthermore, aging is associated with hypermethylation in skeletal muscle tissue (Zykovich et al., 2014; Turner et al., 2020) and muscle stem cells (Turner et al., 2020). With increased physical activity and resistance exercise demonstrated to partially reverse the hypermethylated signature observed with age (Blocquiaux et al., 2020; Turner et al., 2020). Therefore, as COD exercise evokes greater hypomethylation than ST running, COD exercise may also have a beneficial epigenomic impact in reversing the hypermethylation observed in skeletal muscle tissue of aged individuals. Indeed, re-interrogation of the methylome in aged skeletal muscle tissues, reveals one of the same *VEGFA* CpG sites (cg04629501) identified in the current study, is hypermethylated with aging (Turner et al., 2020), compared with the hypomethylated response seen with exercise in the present study. Something, that requires future investigation with COD exercise in elderly populations.

Despite COD exercise evoking a larger hypomethylation response versus ST running. One of the surprising findings was the methylome response identified in the exercise alone analysis (both exercise trials pooled and analyzed over 30 min and 24 h). Data suggested that there was a hypermethylated response 30 min post exercise, yet after 24 h, a large majority of the same DMPs were hypomethylated, suggesting an oscillation in the methylation status of these DMPs. Future work could perhaps investigate changes in DNA methyltransferase activity at the protein level, such as DNMT3A and DNMT3B to investigate whether this ‘transient methylation’ phenomena is occurring. However, it is worth mentioning that the effect of the different trials (COD vs. ST) are not included in the exercise alone analysis. Therefore, the significant differences identified between the trials in terms of movement patterns, physiological and metabolic loads may contribute to this finding, and therefore the two-way ANOVA analysis combining trial and time is likely to provide a more meaningful comparison.

At the individual gene level, we identified that Nuclear Receptor Subfamily 4 Group A Member 1 (*NR4A1*), also known as nerve growth factor IB (NGFIB) or NUR77, was hypomethylated in its promoter region in COD vs. ST exercise trials. NR4A1 is also associated with GO ‘protein binding’ and KEGG ‘MAPK signaling,’ pathways also identified to contain enriched hypomethylation at the pathway level in COD vs. ST trials. NR4A1 is member of the steroid-thyroid hormone-retinoid receptor superfamily, where the encoded protein acts as a nuclear transcription factor. NR4A1 has been shown to be increased in response to various signaling events including; calcium, cAMP, growth factors, mechanical stress and cytokines (Maxwell and Muscat, 2006). NR4A1 is the most abundant nuclear receptor in skeletal muscle when compared to NR4A2 and 3, and *NR4A1* expression is higher in fast-twitch muscle (Chao et al., 2007). NR4A1 abundance at the protein level increases with beta-adrenergic stimulation and after exercise (Mahoney et al., 2005; Chao et al., 2007; Wu et al., 2007; Myers et al., 2009; Catoire et al., 2012). NR4A1 is also associated with glucose uptake, glycolysis and glycogenolysis (Chao et al., 2007), and its global knock-out is associated with an increased predisposition to obesity, insulin resistance and reduced muscle mass (Chao et al., 2009; Tontonoz et al., 2015). Overexpression of NR4A1 in skeletal muscle increases oxidative metabolism (Chao et al., 2012). *NR4A1* is also significantly upregulated (0.94 log fold) in exercise-transcriptome meta-analyses (Pillon et al., 2020) and within the top ten genes upregulated in the sprint transcriptome (Rundqvist et al., 2019) used in the comparative transcriptome analysis in the present study. *NR4A1* expression was also ranked second highest in its significant positive correlation with its other family member, *NR4A3* (*r* = 0.72), in a meta-analysis of exercise transcriptome data sets [metamex.eu (Pillon et al., 2020)]. Indeed, *NR4A3* was the most significantly altered gene across all meta-analyses data sets and has been confirmed to be responsive to exercise stimuli *in vitro* (Pillon et al., 2020). In the present study we also confirmed a robust 165-fold increase in *NR4A3* in COD vs. ST trial at the gene expression level. Finally, *NR4A1* methylation has also been linked to epigenetic memory of skeletal muscle in the offspring of high-fat fed mothers and that the memory could be reversed if the offspring undertook exercise (Kasch et al., 2018). Given that hypomethylation can be retained after acute resistance exercise and training (Seaborne et al., 2018a), and given the association of *NR4A1* with an epigenetic memory of nutrient stress, this suggests that the role of *NR4A1* in an epigenetic memory of aerobic exercise in adult human skeletal muscle requires future investigation.

Finally, we also demonstrate that Vascular Endothelial Growth Factor A (*VEGF*), a potent angiogenic factor originally described in vascular endothelial cells (also expressed in skeletal muscle), has enriched hypomethylation in its promoter, close to its transcription start site, in COD versus ST exercise. VEGFA is involved in exercise training-induced capillary growth (Lloyd et al., 2005; Olfert et al., 2010; Delavar et al., 2014) and is increased after acute exercise (Breen et al., 1996; Richardson et al., 1999; Gustafsson and Sundberg, 2000), with protein levels elevated during the first 4 weeks of exercise training (Gustafsson et al., 2002; Hoier et al., 2012). However, levels are lower in muscle of elderly individuals, yet enhanced with training (Croley et al., 2005; Gliemann et al., 2014). In the present study, we also confirmed a larger increase in *VEGFA* gene expression at 24 h in COD vs. ST trials. *VEGFA* was also ranked top (first) most significantly correlated gene at the expression level with the canonical exercise/metabolic regulators *PPARGC1A* (*r* = 0.84) and *NR4A3* (*r* = 0.74) that was also the most significantly upregulated gene across all modes of exercise in Pillon et al. (2020) across 66 published exercise transcriptome data sets. Re-analysis of the meta-analysis transcriptome to match our study design (acute exercise 30 min up to 24 h post in healthy skeletal muscle), also identified significantly positive correlations between *VEGFA*, PPARGC1A (that encodes *PGC1*-α) and *NR4A3* gene expression. We also demonstrated an increase, of a large magnitude in *PPARGC1A* (11.6 vs. 3.8-fold), as well as in *NR4A3* (165.2 vs. 6.8-fold) in COD vs. ST trials. VEGF mediates the upregulation of *NR4A1* via activation of the PKD/HDAC7/MEF2 pathway (Ha et al., 2008; Wang et al., 2008) and addition of VEGF to endothelial cells increases *NR4A1* 30-fold (Ismail et al., 2012). Therefore, to the best of our knowledge, this is the first study to demonstrate that both *VEGFA* and *NR4A1* are epigenetically modified (hypomethylated) and associated with an increase in gene expression after acute exercise. This hypomethylation effect is enhanced in *VEGFA* specifically after COD exercise compared with ST exercise. Our data raises the possibility that repeated COD exercise may improve VEGFs regulation of capillary formation in response to exercise. Therefore, future studies should undertake repeated exercise (training) with COD versus ST exercise in order to confirm whether the enhanced epigenetic change in VEGF leads to improved capillary formation.

It is important to note while there was a smaller cohort used in the present study for the discovery of differentially methylated sites, the significant overlap of this methylome analysis with recent sprint transcriptome datasets and across transcriptome meta-analyses strengthens the findings and the potential applicability of the results to larger cohorts.

## Conclusion

We provide evidence that COD running exercise preferentially hypomethylates the skeletal muscle methylome when compared with ST running exercise. Specifically, we found hypomethylation and increased gene expression of metabolic and angiogenic genes and pathways. The implication of this data suggests that introducing COD into high intensity running protocols could serve as an important modulator of a favorable epigenomic and transcriptomic landscape in response to exercise and trigger greater skeletal muscle remodeling through enhanced gene expression.

## Data Availability Statement

The original contributions presented in the study are publicly available. This data can be found here: GSE162288. Furthermore, all analyzed DNA methylation raw data outputs can be found in the Supplementary Material.

## Ethics Statement

The studies involving human participants were reviewed and approved by Liverpool John Moores University Ethics Committee (ref. 19/SPS/028). The patients/participants provided their written informed consent to participate in this study.

## Author Contributions

M-FM, AH, BD, and AS conceived and designed the research. All authors were involved in acquisition or analysis or interpretation of data for the work. M-FM, BD, and AS drafted the work. All authors were involved in revising the work critically for important intellectual content. All authors approved the final version of the manuscript.

## Conflict of Interest

The authors declare that the research was conducted in the absence of any commercial or financial relationships that could be construed as a potential conflict of interest.

## References

[B1] Barres R. Yan J. Egan B. Treebak J. T. Rasmussen M. Fritz T. (2012). Acute exercise remodels promoter methylation in human skeletal muscle. *Cell Metab* 15 405–411. 10.1016/j.cmet.2012.01.001 22405075

[B2] Blocquiaux S. Ramaekers M. Van Thienen R. Nielens H. Delecluse C. De Bock K. (2020). Recurrent training rejuvenates and enhances transcriptome and methylome responses in young and older human muscle. *bioRxiv [Preprint]* 10.1101/2020.1106.1130.179465

[B3] Bogdanovic O. Veenstra G. J. (2009). DNA methylation and methyl-CpG binding proteins: developmental requirements and function. *Chromosoma* 118 549–565. 10.1007/s00412-009-0221-9 19506892 PMC2729420

[B4] Breen E. C. Johnson E. C. Wagner H. Tseng H. M. Sung L. A. Wagner P. D. (1996). Angiogenic growth factor mRNA responses in muscle to a single bout of exercise. *J. Appl. Physiol.* 81 355–361. 10.1152/jappl.1996.81.1.355 8828685

[B5] Catoire M. Mensink M. Boekschoten M. V. Hangelbroek R. Müller M. Schrauwen P. (2012). Pronounced effects of acute endurance exercise on gene expression in resting and exercising human skeletal muscle. *PLoS One* 7:e51066. 10.1371/journal.pone.0051066 23226462 PMC3511348

[B6] Chao L. C. Wroblewski K. Ilkayeva O. R. Stevens R. D. Bain J. Meyer G. A. (2012). Skeletal muscle Nur77 expression enhances oxidative metabolism and substrate utilization. *J. Lipid Res.* 53 2610–2619. 10.1194/jlr.m029355 23028113 PMC3494265

[B7] Chao L. C. Wroblewski K. Zhang Z. Pei L. Vergnes L. Ilkayeva O. R. (2009). Insulin resistance and altered systemic glucose metabolism in mice lacking Nur77. *Diabetes* 58 2788–2796. 10.2337/db09-0763 19741162 PMC2780886

[B8] Chao L. C. Zhang Z. Pei L. Saito T. Tontonoz P. Pilch P. F. (2007). Nur77 coordinately regulates expression of genes linked to glucose metabolism in skeletal muscle. *Mol. Endocrinol.* 21 2152–2163. 10.1210/me.2007-0169 17550977 PMC2602962

[B9] Croley A. N. Zwetsloot K. A. Westerkamp L. M. Ryan N. A. Pendergast A. M. Hickner R. C. (2005). Lower capillarization, VEGF protein, and VEGF mRNA response to acute exercise in the vastus lateralis muscle of aged vs. young women. *J. Appl. Physiol.* 99 1872–1879. 10.1152/japplphysiol.00498.2005 16024519

[B10] Delavar H. Nogueira L. Wagner P. D. Hogan M. C. Metzger D. Breen E. C. (2014). Skeletal myofiber VEGF is essential for the exercise training response in adult mice. *Am. J. Physiol. Regul. Integr. Comp. Physiol.* 306 R586–R595. 24523345 10.1152/ajpregu.00522.2013 PMC4043130

[B11] Du P. Zhang X. Huang C.-C. Jafari N. Kibbe W. A. Hou L. (2010). Comparison of beta-value and M-value methods for quantifying methylation levels by microarray analysis. *BMC Bioinformatics* 11:587. 10.1186/1471-2105-11-587 21118553 PMC3012676

[B12] Gliemann L. Olesen J. Biensø R. S. Schmidt J. F. Akerstrom T. Nyberg M. (2014). Resveratrol modulates the angiogenic response to exercise training in skeletal muscles of aged men. *Am. J. Physiol. Heart Circ. Physiol.* 307 H1111–H1119. 25128170 10.1152/ajpheart.00168.2014

[B13] Gustafsson T. Knutsson A. Puntschart A. Kaijser L. Nordqvist A. C. Sundberg C. J. (2002). Increased expression of vascular endothelial growth factor in human skeletal muscle in response to short-term one-legged exercise training. *Pflugers Arch.* 444 752–759. 10.1007/s00424-002-0845-6 12355175

[B14] Gustafsson T. Sundberg C. J. (2000). Expression of angiogenic growth factors in human skeletal muscle in response to a singular bout of exercise. *Am. J. Physiol. Heart Circ. Physiol.* 279 H3144–H3145. 11186876 10.1152/ajpheart.2000.279.6.H3146

[B15] Ha C. H. Jhun B. S. Kao H. Y. Jin Z. G. (2008). VEGF stimulates HDAC7 phosphorylation and cytoplasmic accumulation modulating matrix metalloproteinase expression and angiogenesis. *Arterioscler. Thromb. Vasc. Biol.* 28 1782–1788. 10.1161/atvbaha.108.172528 18617643 PMC2746922

[B16] Handschin C. Chin S. Li P. Liu F. Maratos-Flier E. Lebrasseur N. K. (2007). Skeletal muscle fiber-type switching, exercise intolerance, and myopathy in PGC-1alpha muscle-specific knock-out animals. *J. Biol. Chem.* 282 30014–30021. 10.1074/jbc.m704817200 17702743

[B17] Hoier B. Nordsborg N. Andersen S. Jensen L. Nybo L. Bangsbo J. (2012). Pro- and anti-angiogenic factors in human skeletal muscle in response to acute exercise and training. *J. Physiol.* 590 595–606. 10.1113/jphysiol.2011.216135 22155930 PMC3379703

[B18] Hughes D. C. Turner D. C. Baehr L. M. Seaborne R. A. Viggars M. Jarvis J. C. (2021). Knockdown of the E3 ubiquitin ligase UBR5 and its role in skeletal muscle anabolism. *Am. J. Physiol. Cell Physiol.* 320, C45–C56. 10.1152/ajpcell.00432.2020 33052072

[B19] Ismail H. Mofarrahi M. Echavarria R. Harel S. Verdin E. Lim H. W. (2012). Angiopoietin-1 and vascular endothelial growth factor regulation of leukocyte adhesion to endothelial cells: role of nuclear receptor-77. *Arterioscler. Thromb. Vasc. Biol.* 32 1707–1716. 10.1161/atvbaha.112.251546 22628435 PMC4183139

[B20] Kanehisa M. Furumichi M. Tanabe M. Sato Y. Morishima K. (2017). KEGG: new perspectives on genomes, pathways, diseases and drugs. *Nucleic Acids Res.* 45 D353–D361. 27899662 10.1093/nar/gkw1092 PMC5210567

[B21] Kanehisa M. Goto S. (2000). KEGG: kyoto encyclopedia of genes and genomes. *Nucleic Acids Res.* 28 27–30. 10592173 10.1093/nar/28.1.27 PMC102409

[B22] Kanehisa M. Sato Y. Kawashima M. Furumichi M. Tanabe M. (2016). KEGG as a reference resource for gene and protein annotation. *Nucleic Acids Res.* 44 D457–D462. 26476454 10.1093/nar/gkv1070 PMC4702792

[B23] Kasch J. Kanzleiter I. Saussenthaler S. Schurmann A. Keijer J. Van Schothorst E. (2018). Insulin sensitivity linked skeletal muscle Nr4a1 DNA methylation is programmed by the maternal diet and modulated by voluntary exercise in mice. *J. Nutr. Biochem.* 57 86–92. 10.1016/j.jnutbio.2018.03.015 29680662

[B24] Lindholm M. E. Marabita F. Gomez-Cabrero D. Rundqvist H. Ekström T. J. Tegnér J. (2014). An integrative analysis reveals coordinated reprogramming of the epigenome and the transcriptome in human skeletal muscle after training. *Epigenetics* 9 1557–1569. 10.4161/15592294.2014.982445 25484259 PMC4622000

[B25] Lister R. Pelizzola M. Dowen R. H. Hawkins R. D. Hon G. Tonti-Filippini J. (2009). Human DNA methylomes at base resolution show widespread epigenomic differences. *Nature* 462 315–322. 10.1038/nature08514 19829295 PMC2857523

[B26] Liu J.-X. Zhu L. Li P.-J. Li N. Xu Y.-B. (2019). Effectiveness of high-intensity interval training on glycemic control and cardiorespiratory fitness in patients with type 2 diabetes: a systematic review and meta-analysis. *Aging Clin. Exp. Res.* 31 575–593. 10.1007/s40520-018-1012-z 30097811 PMC6491404

[B27] Lloyd P. G. Prior B. M. Li H. Yang H. T. Terjung R. L. (2005). VEGF receptor antagonism blocks arteriogenesis, but only partially inhibits angiogenesis, in skeletal muscle of exercise-trained rats. *Am. J. Physiol. Heart Circ. Physiol.* 288 H759–H768. 15471974 10.1152/ajpheart.00786.2004

[B28] Maasar M. F. Turner D. C. Gorski P. P. Seaborne R. A. Strauss J. A. Shepherd S. O. (2020). The methylome and comparative transcriptome after high intensity sprint exercise in human skeletal muscle. *bioRxiv [Preprint]*

[B29] Mahoney D. J. Parise G. Melov S. Safdar A. Tarnopolsky M. A. (2005). Analysis of global mRNA expression in human skeletal muscle during recovery from endurance exercise. *Faseb. J.* 19 1498–1500. 10.1096/fj.04-3149fje 15985525

[B30] Maksimovic J. Gordon L. Oshlack A. (2012). SWAN: subset-quantile within array normalization for illumina infinium HumanMethylation450 BeadChips. *Genome Biol.* 13:R44. 10.1186/gb-2012-13-6-r44 PMC3446316 22703947

[B31] Maksimovic J. Phipson B. Oshlack A. (2016). A cross-package Bioconductor workflow for analysing methylation array data [version 1; referees: 3 approved, 1 approved with reservations]. *F1000Res.* 5:1281. 10.12688/f1000research.8839.1 PMC4916993 27347385

[B32] Maxwell M. A. Muscat G. E. (2006). The NR4A subgroup: immediate early response genes with pleiotropic physiological roles. *Nucl. Recept. Signal.* 4:e002. 10.1621/nrs.04002 PMC1402209 16604165

[B33] Myers S. A. Eriksson N. Burow R. Wang S. C. Muscat G. E. (2009). Beta-adrenergic signaling regulates NR4A nuclear receptor and metabolic gene expression in multiple tissues. *Mol. Cell Endocrinol.* 309 101–108. 10.1016/j.mce.2009.05.006 19465082

[B34] Nicolella D. P. Torres-Ronda L. Saylor K. J. Schelling X. (2018). Validity and reliability of an accelerometer-based player tracking device. *PLoS One* 13:e0191823. 10.1371/journal.pone.0191823 29420555 PMC5805236

[B35] Nitert M. D. Dayeh T. Volkov P. Elgzyri T. Hall E. Nilsson E. (2012). Impact of an exercise intervention on DNA methylation in skeletal muscle from first-degree relatives of patients with type 2 diabetes. *Diabetes* 61 3322–3332. 10.2337/db11-1653 23028138 PMC3501844

[B36] Olfert I. M. Howlett R. A. Wagner P. D. Breen E. C. (2010). Myocyte vascular endothelial growth factor is required for exercise-induced skeletal muscle angiogenesis. *Am. J. Physiol. Regul. Integr. Comp. Physiol.* 299 R1059–R1067. 20686173 10.1152/ajpregu.00347.2010 PMC2957383

[B37] PatelH.SyddallH. E.MartinH. J.CooperC.StewartC.SayerA. A. (2011). The feasibility and acceptability of muscle biopsy in epidemiological studies: findings from the hertfordshire sarcopenia study (HSS). *J. Nutr. Health Aging* 15 10–15. 10.1007/s12603-011-0006-8 21267515

[B38] Pidsley R. Zotenko E. Peters T. J. Lawrence M. G. Risbridger G. P. Molloy P. (2016). Critical evaluation of the Illumina MethylationEPIC BeadChip microarray for whole-genome DNA methylation profiling. *Genome Biol.* 17:208. 10.1186/s13059-016-1066-1 PMC5055731 27717381

[B39] Pillon N. J. Gabriel B. M. Dollet L. Smith J. A. B. Sardón Puig L. Botella J. (2020). Transcriptomic profiling of skeletal muscle adaptations to exercise and inactivity. *Nat. Commun.* 11:470. 10.1038/s41467-019-13869-w PMC6981202 31980607

[B40] Richardson R. S. Wagner H. Mudaliar S. R. Henry R. Noyszewski E. A. Wagner P. D. (1999). Human VEGF gene expression in skeletal muscle: effect of acute normoxic and hypoxic exercise. *Am. J. Physiol.* 277 H2247–H2252. 10600843 10.1152/ajpheart.1999.277.6.H2247

[B41] Rowlands D. S. Page R. A. Sukala W. R. Giri M. Ghimbovschi S. D. Hayat I. (2014). Multi-omic integrated networks connect DNA methylation and miRNA with skeletal muscle plasticity to chronic exercise in Type 2 diabetic obesity. *Physiol. Genomics* 46 747–765. 10.1152/physiolgenomics.00024.2014 25138607 PMC4200377

[B42] Rundqvist H. C. Montelius A. Osterlund T. Norman B. Esbjornsson M. Jansson E. (2019). Acute sprint exercise transcriptome in human skeletal muscle. *PLoS One* 14:e0223024. 10.1371/journal.pone.0223024 31647849 PMC6812755

[B43] Schmittgen T. D. Livak K. J. (2008). Analyzing real-time PCR data by the comparative C(T) method. *Nat. Protoc.* 3 1101–1108. 10.1038/nprot.2008.73 18546601

[B44] Seaborne R. A. Hughes D. C. Turner D. C. Owens D. J. Baehr L. M. Gorski P. (2019). UBR5 is a novel E3 ubiquitin ligase involved in skeletal muscle hypertrophy and recovery from atrophy. *J. Physiol.* 597 3727–3749. 10.1113/jp278073 31093990

[B45] Seaborne R. A. Strauss J. Cocks M. Shepherd S. O’brien T. D. Someren K. A. V. (2018a). Human skeletal muscle possesses an epigenetic memory of hypertrophy. *Sci. Rep.* 8:1898. 10.1038/s41598-018-20287-3 PMC5789890 29382913

[B46] Seaborne R. A. Strauss J. Cocks M. Shepherd S. O’brien T. D. Someren K. A. V. (2018b). Methylome of human skeletal muscle after acute & chronic resistance exercise training, detraining & retraining. *Sci. Data* 5:180213. 10.1038/sdata.2018.213 PMC6207066 30375987

[B47] Stephens N. A. Brouwers B. Eroshkin A. M. Yi F. Cornnell H. H. Meyer C. (2018). Exercise response variations in skeletal muscle PCr recovery rate and insulin sensitivity relate to muscle epigenomic profiles in individuals with type 2 diabetes. *Diabetes Care* 41 2245–2254. 10.2337/dc18-0296 30072402

[B48] Tontonoz P. Cortez-Toledo O. Wroblewski K. Hong C. Lim L. Carranza R. (2015). The orphan nuclear receptor Nur77 is a determinant of myofiber size and muscle mass in mice. *Mol. Cell Biol.* 35 1125–1138. 10.1128/mcb.00715-14 25605333 PMC4355536

[B49] Turner D. C. Gorski P. P. Maasar M. F. Seaborne R. A. Baumert P. Brown A. D. (2020). DNA methylation across the genome in aged human skeletal muscle tissue and muscle-derived cells: the role of HOX genes and physical activity. *Sci. Rep.* 10:15360. 10.1038/s41598-020-72730-z 32958812 PMC7506549

[B50] Turner D. C. Seaborne R. A. Sharples A. P. (2019). Comparative transcriptome and methylome analysis in human skeletal muscle anabolism, hypertrophy and epigenetic memory. *Sci. Rep.* 9:4251. 10.1038/s41598-019-40787-0 PMC6414679 30862794

[B51] Wang S. Li X. Parra M. Verdin E. Bassel-Duby R. Olson E. N. (2008). Control of endothelial cell proliferation and migration by VEGF signaling to histone deacetylase 7. *Proc. Natl. Acad. Sci. U.S.A.* 105 7738–7743. 10.1073/pnas.0802857105 18509061 PMC2409381

[B52] Weston M. Siegler J. Bahnert A. Mcbrien J. Lovell R. (2015). The application of differential ratings of perceived exertion to Australian Football League matches. *J. Sci. Med. Sport* 18 704–708. 10.1016/j.jsams.2014.09.001 25241705

[B53] Wu X. Wang J. Cui X. Maianu L. Rhees B. Rosinski J. (2007). The effect of insulin on expression of genes and biochemical pathways in human skeletal muscle. *Endocrine* 31 5–17. 10.1007/s12020-007-0007-x 17709892

[B54] Ziller M. J. Gu H. Muller F. Donaghey J. Tsai L. T. Kohlbacher O. (2013). Charting a dynamic DNA methylation landscape of the human genome. *Nature* 500 477–481. 10.1038/nature12433 23925113 PMC3821869

[B55] Zykovich A. Hubbard A. Flynn J. M. Tarnopolsky M. Fraga M. F. Kerksick C. (2014). Genome-wide DNA methylation changes with age in disease-free human skeletal muscle. *Aging Cell* 13 360–366. 10.1111/acel.12180 24304487 PMC3954952

