# Supplementary figures and images for: The Comparative Methylome and Transcriptome After Change of Direction Compared to Straight Line Running Exercise in Human Skeletal Muscle
Source: Front Physiol. 2021 Feb 19;12:619447. doi: 10.3389/fphys.2021.619447 (PMC7933519; doi:10.3389/fphys.2021.619447)

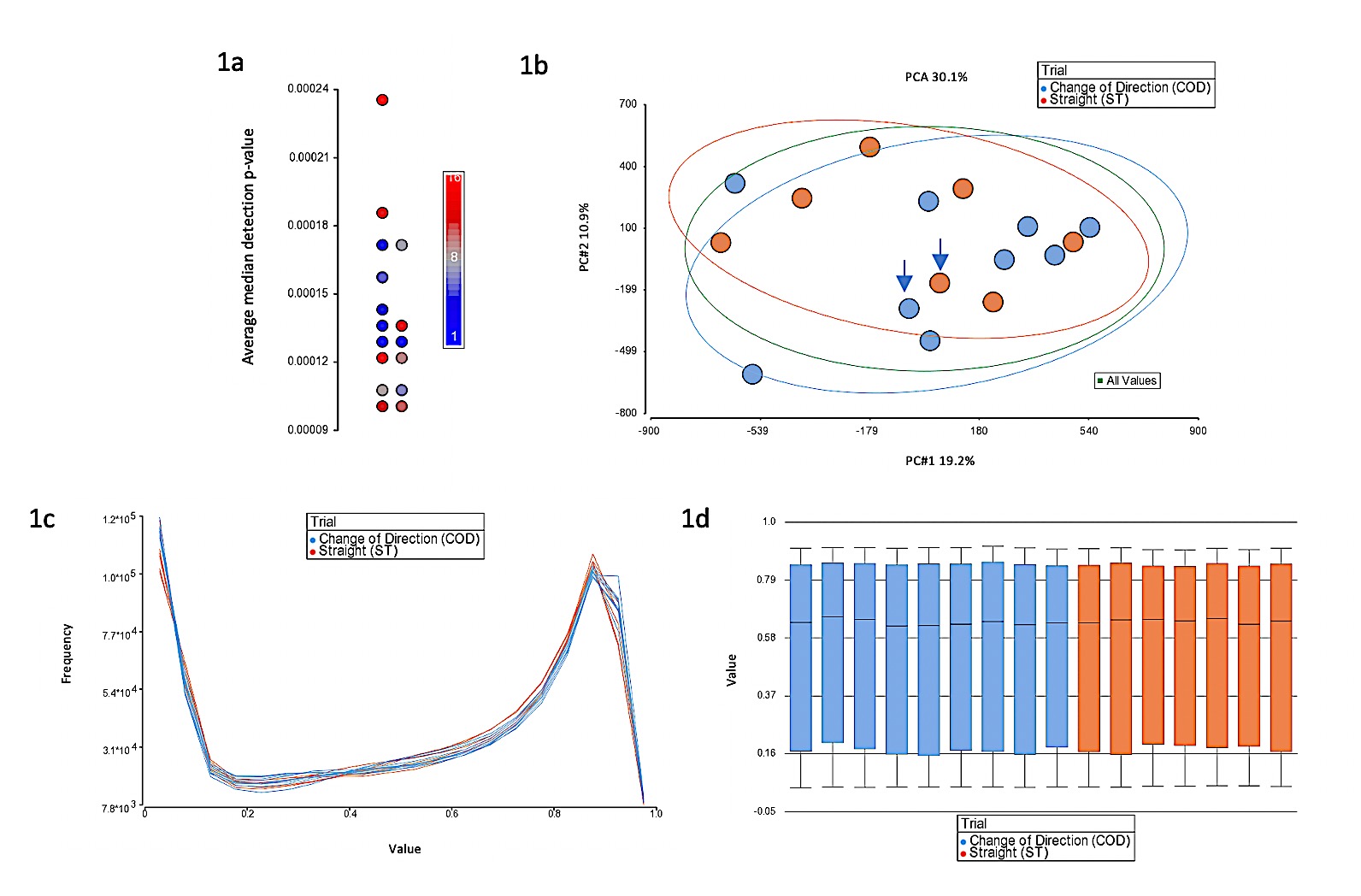

Supplement: Supplementary file 9 [file Image_1.JPEG]
